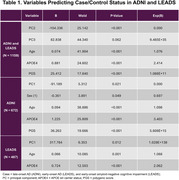# Polygenic Alzheimer's Disease Risk in Early‐ and Late‐Onset Alzheimer's Disease

**DOI:** 10.1002/alz70855_107414

**Published:** 2025-12-25

**Authors:** Julian V. Pentchev, Trever Jackson, Thea Jacobson Rosewood, Yen‐Ning Huang, Kwangsik Nho, Andrew J. Saykin, Ani Eloyan, Jeffrey L. Dage, Tatiana M. Foroud, Dustin B. Hammers, Maria C. Carrillo, Brad C. Dickerson, Gil D. Rabinovici, Liana G. Apostolova, Kelly N. Nudelman, LEADS Consortium

**Affiliations:** ^1^ Indiana University, Bloomington, IN, USA; ^2^ Department of Medical and Molecular Genetics, Indiana University School of Medicine, Indianapolis, IN, USA; ^3^ Department of Radiology and Imaging Sciences, IUSM, Indianapolis, IN, USA; ^4^ Department of Medical and Molecular Genetics, Indianapolis, IN, USA; ^5^ Indiana Alzheimer's Disease Research Center, Indianapolis, IN, USA; ^6^ Center for Neuroimaging, Department of Radiology and Imaging Sciences, Indiana University School of Medicine, Indianapolis, IN, USA; ^7^ Indiana University School of Informatics and Computing, Indianapolis, IN, USA; ^8^ Center for Neuroimaging, Department of Radiology and Imaging Sciences, Indiana University School of Medicine, Indianapolis, IN, USA; ^9^ Indiana Alzheimer's Disease Research Center, Indiana University School of Medicine, Indianapolis, IN, USA; ^10^ Center for Neuroimaging, Indiana University School of Medicine, Indianapolis, IN, USA; ^11^ Department of Biostatistics, Brown University, Providence, RI, USA; ^12^ Stark Neurosciences Research Institute, Indiana University School of Medicine, Indiana, IN, USA; ^13^ Department of Neurology, Indiana University School of Medicine, Indianopolis, IN, USA; ^14^ Stark Neurosciences Research Institute, Indiana University School of Medicine, Indianapolis, IN, USA; ^15^ Indiana University School of Medicine, Indianapolis, IN, USA; ^16^ Medical & Scientific Relations Division, Alzheimer's Association, Chicago, IL, USA; ^17^ Frontotemporal Disorders Unit and Massachusetts Alzheimer's Disease Research Center, Department of Neurology, Massachusetts General Hospital and Harvard Medical School, Boston, MA, USA; ^18^ Memory and Aging Center, University of California San Francisco, San Francisco, CA, USA; ^19^ Memory and Aging Center, Weill Institute for Neurosciences, University of California, San Francisco (UCSF), San Francisco, CA, USA; ^20^ Department of Neurology, Indiana University School of Medicine, Indianapolis, IN, USA; ^21^ Indiana Alzheimer's Disease Research Center, Indiana University School of Medicine, Indianapolis, IN, USA; ^22^ Department of Radiology and Imaging Sciences, Center for Neuroimaging, Indiana University School of Medicine, Indianapolis, IN, USA

## Abstract

**Background:**

Most of the genetic etiology for early‐onset Alzheimer's disease (EOAD) is currently unexplained. One hypothesis is that EOAD cases may have similar genetic etiology to late‐onset AD (LOAD), but with more LOAD risk SNPs than typical LOAD patients, reflected by higher polygenic scores (PGS) and earlier age of onset (AoO). We will compare the predictive value of a LOAD PGS for EOAD and LOAD in the Longitudinal Early Onset Alzheimer's Disease Study (LEADS) and the Alzheimer's Disease Neuroimaging Initiative (ADNI).

**Method:**

A PGS developed by Desikan et al., 2017 was applied to GWAS data for white non‐Hispanic harmonized LEADS (*N* = 487) and ADNI (*N* = 672) participants. Binary logistic regression models were tested to identify predictors of EOAD and LOAD. In the combined cohort, this model was also run covarying for APOE4 carrier status. Cox regression was used to assess differences in ADNI and LEADS AoO for tertile‐binned PGS. Within LEADS EOAD participants, five cognitive domains were assessed for correlation with PGS.

**Result:**

The AD PGS predicted case/control status in the combined cohort (X^2^(4)=226.365, *p* <0.001), in the ADNI‐only cohort (X^2^(4)=149.695, *p* = <0.001), and in the LEADS‐only cohort (X^2^(3)=30.323, *p* <0.001). In each model, higher PGS was associated with increased odds of EOAD and/or LOAD. Covarying for APOE4, PGS remained significant in the combined cohort (X^2^(5)=250.904, *p* <0.001) and in the ADNI cohort (X^2^(5)=176.307, *p* <0.001) but not in the LEADS‐only cohort (X^2^(3)=31.974, *p* = 0<0.001; final model did not include PGS) (Table 1). Hazard survival analysis indicated a significant difference in PGS groups in the combined and ADNI cohorts’ AoO but not in the LEADS cohort. In the EOAD group, PGS was significantly correlated with cognitive score for visuospatial performance (F=4.473 *p* = 0.013). Language performance was also correlated with PGS in EOAD (F=3.387 *p* = 0.036).

**Conclusion:**

These results suggest that LOAD genetic risk, aside from APOE4, is not a strong driver of EOAD age of onset; however, these variants may mediate some aspects of cognitive dysfunction in EOAD patients.